# The Interrelationship Between Motor Coordination and Adaptive Behavior in Children With Autism Spectrum Disorder

**DOI:** 10.3389/fpsyg.2018.02350

**Published:** 2018-11-27

**Authors:** Emily Bremer, John Cairney

**Affiliations:** ^1^Department of Kinesiology, McMaster University, Hamilton, ON, Canada; ^2^Department of Family Medicine, McMaster University, Hamilton, ON, Canada; ^3^Faculty of Kinesiology and Physical Education, University of Toronto, Toronto, ON, Canada

**Keywords:** motor development, neurodevelopmental disability, middle childhood, developmental trajectories, daily living skills

## Abstract

**Objective:** Children with autism spectrum disorder (ASD) experience significant challenges with their motor coordination. It is not, however, well understood how motor coordination may impact the behavioral functioning of children with ASD. Therefore the purpose of this study was to explore the relationships between motor coordination and adaptive behavior in 7–12-year-old children with ASD.

**Methods:** Motor coordination was assessed using the Movement Assessment Battery for Children, 2nd Edition (MABC-2) and adaptive behavior was assessed by parental report using the Vineland Adaptive Behavior Scales, 2nd Edition (VABS-2) as part of a larger cross-sectional study. Descriptive characteristics were calculated for MABC-2 and VABS-2 scores and Spearman’s rank order correlation analyses were used to examine the relationship between motor coordination and adaptive behavior.

**Results:** On average, the participants (*n* = 26) exhibited significant challenges in regard to their motor coordination with all but two participants classified as having significant motor impairments by scoring at or below the 16th percentile on the MABC-2. Results from the correlation analyses indicated that manual dexterity was positively related to daily living skills (ρ = 0.58, *p* < 0.003), and overall motor coordination was positively related to daily living skills (ρ = 0.60, *p* < 0.003) and overall adaptive behavior (ρ = 0.57, *p* < 0.003). In all instances, better motor coordination was related to more adaptive behaviors.

**Conclusion:** These results highlight the profound motor coordination challenges that children with ASD experience and also suggest that these challenges, particularly with manual dexterity, are related to the daily behavior of children with ASD. The interrelatedness of motor and adaptive behavior suggests the need to further explore the impact of motor-based interventions for this population, as well as conduct longitudinal studies to disentangle these relationships.

## Introduction

Autism spectrum disorder (ASD) is a neurodevelopmental disorder defined by challenges in communication, social skills, and the presence of restricted and repetitive behaviors (American Psychiatric Association, 2013). Children with ASD are heterogeneous in their abilities and difficulties, with varying levels of symptom severity, behavioral profiles, and relative level of functioning for each individual child with ASD (Geschwind, 2009; Wiggins et al., 2012; Georgiades et al., 2013a,b). Adaptive behavior is a common term used to describe and classify the degree to which an individual performs daily activities for social and personal sufficiency and for school-aged children this can include tasks such as household chores, completing school projects, and getting along with peers (Sparrow et al., 2005). Children with ASD are most commonly described as having adaptive behavior profiles with relative strengths in daily living skills, but with weaknesses in socialization and communication (Kanne et al., 2011). Adaptive behavior is consistently associated with positive outcome in individuals with ASD, regardless of symptom severity (Kanne et al., 2011), further emphasizing the importance of adaptive behavior in enabling individuals with ASD to reach a level of independence needed for personal and social sufficiency.

One area of development that is often ignored when intervening on the adaptive behavior of school-aged children with ASD is their motor coordination. Motor coordination is the organization of one’s body parts to create both gross and fine movements (Payne and Isaacs, 2011; Haywood and Getchell, 2014). Motor coordination is involved in virtually all aspects of daily living including, fine motor tasks like dressing, feeding, and writing and gross motor tasks including locomotion and active play. Beyond basic motor tasks, motor coordination is inherently linked to social communicative skills through gesturing, imitation, and joint attention (Schmidt et al., 2011); skills that are typically a challenge for children with ASD. Motor delays are present early on for toddlers with ASD and this delay may become more pronounced with age (Lloyd et al., 2013). Importantly, these motor delays are not limited to infants and toddlers, but persist through childhood into adulthood (Ozonoff et al., 2008; Fournier et al., 2010; Bhat et al., 2011). For example, the most recent meta-analysis of 51 studies showed that individuals with ASD had significantly poorer motor coordination across a range of domains (e.g., balance, posture, gait, reaction time, aiming) when compared to typically developing peers (Fournier et al., 2010).

Until relatively recently, motor coordination problems in children, characteristic of a disorder known as developmental coordination disorder (DCD) (Cairney, 2015), was considered a separate, non-overlapping, condition from ASD. Indeed, in the case of DCD, until the 5th edition of the Diagnostic and Statistical Manual of Mental Disorders (American Psychiatric Association, 2013), children who met diagnostic criteria for ASD were excluded altogether from a diagnosis of DCD. This reflects a pervasive underlying concern about evaluating a child’s motor ability independent of their cognitive ability. Since communicative disorders are an inherent feature of ASD (American Psychiatric Association, 2013), poor performance on motor tasks may be related to failure to comprehend or comply with verbal instructions, rather than reflecting a true deficit in motor ability. At the same time, it has been observed clinically for some time that children with neurodevelopmental disorders such as ADHD/ADD or ASD, typically present with motor impairments (Cairney and King-Dowling, 2016). Evidence from a recent systematic review suggests that behaviorally DCD and ASD are separate, but possibly co-occurring, conditions with differences in many behavioral domains (Caçola et al., 2017); however, these findings are limited by the lack of studies reporting on children with co-occurring diagnoses of ASD and DCD. Indeed, the removal of ASD as an exclusion from DCD has opened the door for more serious inquiry into co-occurring, motor, cognitive, communicative and social impairments in children who present with neurodevelopmental disorders such as ASD. Examination of these co-occurring conditions helps us to better understand the heterogeneity of ASD, while also providing opportunities to discern appropriate treatment strategies for different subgroups of the spectrum. This is important given the range of behavioral profiles exhibited by children with ASD, along with the notion that co-occurring conditions may actually be the most prominent difficulties in the daily life of a child with ASD (Gillberg and Fernell, 2014).

Despite the evidence that motor impairments are common in children with ASD, there is still relatively little evidence on how these delays may be related to the core deficits of the disorder and the ensuing implications on their adaptive behavior. Previous research has demonstrated that significant relationships exist between motor development, social skills, and communication in infants developing both typically and atypically (Leonard and Hill, 2014). However, the importance of these relationships is often neglected beyond the developmental period in children with ASD. Existing research in school-aged children is limited but suggests that fundamental movement skills (i.e., the foundational skills needed for participation in physical activity) are related to social communicative skills in this population (MacDonald et al., 2013). The relationships between fundamental movement skills and social skills inherently make sense for children with ASD: proficient movement skills are necessary to engage in active play, which in turn is a very important social activity for children. The current literature is limited in that it has yet to explore whether motor coordination, including both gross and fine motor skill, is related to outcomes beyond just social communicative skills in children with ASD, which may have important implications for independence and positive outcomes. Thus, the purpose of this study is to examine the relationship between motor coordination and adaptive behavior in school-aged children with ASD.

## Materials and Methods

### Design and Procedure

A cross-sectional design was employed to assess the relationship between motor coordination and adaptive behavior in 7–12-year-old children with ASD. The study consisted of one appointment, which represented the second visit in a larger study of the physical health of children with ASD. The first study appointment was a lab familiarization session for the participant and their parent; no data collection occurred at this appointment. Participants were recruited on an on-going basis and assessments took place in a university research lab between March 2016 and December 2017. All assessments were conducted by a graduate research assistant with formal training in the assessment of motor development and coordination in children with ASD. Ethical approval for the study was provided by the university’s Institutional Research Ethics Board. Informed written consent was obtained from the participants’ parent or guardian at the first study appointment and participants 8 years of age and older provided informed written assent prior to their participation.

### Participants

A convenience sample of participants were recruited through advertisements at local community centers, in addition to a recruitment mail out to families of children with ASD on a waitlist for government-funded applied behavior analysis services in the geographic region of the study. Children were eligible to participate if they were between the ages of 7 years, 0 months, to 12 years, 11 months with confirmation of their ASD diagnosis provided by their parent or guardian. In Ontario, Canada, where this study took place, a diagnosis of ASD can be made by a family physician, pediatrician, psychiatrist, psychologist, or a psychological associate.

### Measures

#### Demographic and Medical History

The participants’ diagnostic and treatment history was reported by parental completion of a supplemental information form. This form asked parents to answer basic demographic questions, state when (and by whom) their child was diagnosed, and asked a range of questions regarding their child’s motor and behavioral treatment history.

#### Motor Coordination

Children were administered the Movement Assessment Battery for Children-2 (MABC-2) by a trained research assistant, which is an individually administered standardized test used to identify motor impairment in children aged 3–16 years (Henderson et al., 2007). The MABC-2 consists of eight motor tasks grouped under three domains: manual dexterity; aiming & catching; and balance. Domain and total test scores for the assessment are converted into age-normed percentile scores, with a score below the 16th percentile indicating the presence of significant motor impairments. Test re-test reliability and standard error of measurement for the total test scores have been reported to be 0.80 and 1.34, respectively (Henderson et al., 2007).

#### Adaptive Behavior

Parents completed the Vineland Adaptive Behavior Scales-2 (VABS-2) Parent/Caregiver Rating Form (Sparrow et al., 2005). The VABS-2 assesses adaptive behavior in the following domains: communication, daily living skills, socialization, and maladaptive behavior, and helps to provide a broad overview of each child’s level of functioning. Standard scores are reported for the domains of communication, daily living skills, and socialization, in addition to the adaptive behavior composite. VABS-2 standard scores range from 20 to 160, have a mean of 100, and a standard deviation of 15. Maladaptive behaviors are reported for the domains of externalizing and internalizing, in addition to a composite maladaptive behavior index. Each of the maladaptive behavior scores are reported as *v*-scale scores, which range from 1–24 with a mean of 15, and a standard deviation of 3. Internal-consistency reliabilities of the domain and composite scores of the VABS-2 range from 0.80 to 0.97 and the test–retest reliability correlations range from 0.75 to 0.93 for 7–12-year-old children (Sparrow et al., 2005).

### Statistical Analyses

Descriptive statistics (mean, standard deviations) were calculated for demographic and diagnostic variables, along with domain and total scores for the MABC-2 and VABS-2. Tests of normality revealed that the MABC-2 scores were not normally distributed, therefore nonparametric analyses were employed. Spearman’s rank order correlations were used to examine the relationships between MABC-2 and VABS-2 domain and total scores. Given the multiple correlations being tested, a Bonferroni-adjusted *p*-value of 0.003 (0.05/16) was used as the threshold for statistical significance. All analyses were conducted using SPSS 25 (IBM Corporation, 2017).

## Results

Demographic and diagnostic characteristics of the sample (*n* = 26) are presented in Table 1. On average, the participants’ exhibited impaired motor coordination with an average MABC-2 total test percentile score in the 6th percentile. In other words, participants in this sample were scoring, on average, better than only 6% of their age- and sex-matched peers. Moreover, all but two participants were classified as having significant motor impairments by scoring at or below the 16th percentile on the MABC-2 (Table 2). As anticipated, the participants’ also exhibited challenges with their adaptive behavior. For example, the VABS-2 adaptive behavior composite score was, on average, almost two standard deviations below what would be expected for the participants’ age. Likewise, on average, the maladaptive behavior scores ranged from one to almost two standard deviations above what would be expected for the participants’ age, indicating an increased level of maladaptive behavior (Table 2).

**Table 1:** Descriptive characteristics of the sample (*n* = 26).

Variable		Statistics
		Mean (SD)
Age (years)		9.9 (1.7)
Age at diagnosis (months)		60.6 (28.2)
		***N (%)***
Sex (male, female)		*23* male, *3* female
Ethnicity		
	Caucasian	*19 (73)*
	Other	*7 (27)*
Diagnosis provider	
	Pediatrician	*15 (58)*
	Psychiatrist	*3 (12)*
	Psychologist	*7 (27)*
	Psychological associate	*1 (3)*
Previously received treatment	
	Behavioral (ABA or IBI)	*19 (73)*
	Speech-Language	*19 (73)*
	Motor (PT or OT)	*15 (58)*
Currently receiving treatment	
	Behavioral (ABA or IBI)	*10 (38)*
	Speech-language	*4 (15)*
	Motor (PT or OT)	*8 (31)*
Number of co-occurring diagnoses	
	0	*14 (53.8)*
	1	*3 (11.5)*
	2	*6 (23.1)*
	3	*1 (3.8)*
	4	*2 (7.7)*
Type of co-occurring diagnoses	
	ADD/ADHD	*9 (34.6)*
	Developmental coordination disorder	*2 (7.7)*
	Developmental delay	*6 (23.1)*
	Giftedness	*1 (3.8)*
	Intellectual disability	*2 (7.7)*
	Learning disability	*3 (11.5)*
	Selective mutism	*1 (3.8)*
	Tourette syndrome	*2 (7.7)*


*ABA, Applied Behavior Analysis; IBI, Intensive Behavioral Intervention; PT, Physiotherapy; OT, Occupational Therapy; ADD/ADHD, Attention Deficit Disorder/Attention Deficit Hyperactivity Disorder.*

**Table 2:** Motor coordination and adaptive behavior scores.

Variable		Mean (SD)
MABC-2 percentile scores	
	Manual dexterity	3.8 (5.6)
	Aiming & catching	19.4 (27.4)
	Balance	13.5 (22.2)
	Total test	6.3 (13.9)
VABS-2 adaptive behavior standard scores	
	Communication	79.6 (16.2)
	Daily living skills	83.8 (16.4)
	Socialization	77.9 (14.9)
	Adaptive behavior composite	78.9 (13.4)
VABS-2 maladaptive behavior *v*-scale scores	
	Internalizing	20.5 (2.2)
	Externalizing	18.4 (2.4)
	Maladaptive behavior index	20.1 (1.9)


*MABC-2, Movement Assessment Battery for Children, 2nd Edition; VABS-2, Vineland Adaptive Behavior Scales, 2nd Edition.*

Results from the correlation analyses (Table 3) indicate that the manual dexterity domain of the MABC-2 was significantly correlated with the VABS-2 daily living skill domain. Further, the MABC-2 total test score was significantly correlated with the VABS-2 daily living skill domain and the adaptive behavior composite: in all cases, children who performed better on the motor tasks had more adaptive behaviors in these domains. No other significant correlations were present.

**Table 3:** Spearman’s rank order correlations between MABC-2 and VABS-2 scores.

VABS-2	MABC-2			
	Manual dexterity	Aiming & catching	Balance	Total score
Communication	0.34	0.31	0.44	0.42
Daily living skills	0.58^∗^	0.29	0.47	0.60^∗^
Socialization	0.34	0.17	0.34	0.45
Adaptive behavior composite	0.53	0.25	0.45	0.57^∗^


*^∗^Correlation significant at p < 0.003. MABC-2, Movement Assessment Battery for Children, 2nd Edition; VABS-2, Vineland Adaptive Behavior Scales, 2nd Edition.*

## Discussion

The results of this study confirm the presence of significant delays in motor coordination and adaptive behavior experienced by school-aged children with ASD and add to our understanding of how these behavioral domains are related to one another. Specifically, we found that manual dexterity, or fine motor coordination, is strongly related to better functioning in daily living skills and better overall motor coordination was related to better daily living skills and overall adaptive behavior. These findings support previous work that has found positive associations between fundamental movement skills and social communicative skills (MacDonald et al., 2013), and better manual dexterity and overall motor coordination related to less social impairment in children with ASD (Hirata et al., 2014). However, the importance of manual dexterity for daily living skills and more generalized adaptive behavior was previously underexplored in school-aged children with ASD.

Motor development is so closely entwined with a child’s adaptive behavior that fine and gross motor skills are included in the VABS-2 up until the age of 6 years (Sparrow et al., 2005). It is clear however, that the motor domain does not cease to influence adaptive behavior beyond the developmental period, and in fact it may be even more important for school-aged children with ASD as they also exhibit significant motor delays. Hirata et al. (2014) postulate several potential mechanisms linking motor coordination to other areas of behavior and function (specifically social impairments). First, the association may reflect an underlying neurocognitive deficit affecting regions of the brain that govern both motor control, social behavior and cognitive function. Indeed, in the area of DCD, researchers have postulated a pervasive, minimal brain dysfunction as a possible mechanism to explain the high rates of co-morbidity in children with motor coordination problems, particularly in relation to attention and executive functioning (Kaplan et al., 1998; Dewey et al., 2002). The correlation observed here may simply reflect a general disruption in brain function that affects multiple domains simultaneously. For example, both functional and anatomical disturbances in neural connectivity have been implicated in the behavioral characteristics of individuals with ASD (Belmonte, 2004; Schipul et al., 2011), with abnormal cerebellar activity being one common indicator of dysfunction (Belmonte, 2004; Mostofsky et al., 2009). The cerebellum plays an important role in motor development and coordination, and increasing focus is being placed on the interrelationships between the cerebellum and brain regions responsible for cognitive tasks, such as the prefrontal cortex (Diamond, 2000, 2007; Leisman et al., 2016). One could therefore expect that cerebellar dysfunction is not only implicated in the motor impairments associated with ASD but, also in the cognitive and executive dysfunctions associated with the disorder. Moreover, the cerebellum is also responsible for automatization processes, or the ability to complete a task with little conscious attention. The automatization deficit hypothesis, wherein individuals have difficulty automatizing their motor and cognitive behaviors, was initially used as a paradigm for children with developmental dyslexia (Fawcett and Nicolson, 1992), but has more recently been applied to children with DCD (Visser, 2003; Schott et al., 2016). These authors speculate that challenges in automatization resulting from cerebellar dysfunction are part of the neuropathology of DCD and provide rationale for the frequency of co-occurring developmental concerns in areas such as attention and learning (Visser, 2003). It is likely then, that an automatization deficit may also help to explain the motor impairments experienced by children with ASD, in addition to the co-occurrence of social and attentional difficulties.

Another explanation concerns the relationship between manual dexterity specifically and other domains of function (Hirata et al., 2014). It seems reasonable that a child who performs better in manual tasks will also score higher on self-care behaviors and other aspects of daily living, given the importance of these motoric skills for everyday tasks (e.g., combing hair, dressing, eating). With regard to communication, manual dexterity is important for school-based activities (printing, art work), so children with better skills in this domain may also have greater opportunity for developing interpersonal and communicative skills, interacting with teachers and other peers. Of course, it will be necessary to track the longitudinal development of both manual dexterity and adaptive behaviors in children with ASD to untangle possible causal linkages.

It is also important to consider the heterogeneity in ASD and how this may affect movement skill. Previous research has shown mixed results when it comes, for example, to the association between manual dexterity, vs. other domains of motor coordination, and other developmental outcomes (MacDonald et al., 2013; Hirata et al., 2014). Hirata et al. (2014) hypothesize that this in turn may be related to social and/or cultural factors. For example, in some countries, focusing on the development of manual skills through practice may be considered more important by parents than other skills. It is also plausible that the mixed results seen in previous research is simply due to the heterogeneity of the disorder itself. ASD is complex, characterized by behavior, and is often accompanied by co-occurring diagnoses (Gillberg and Fernell, 2014). It is therefore likely that the motor domain will have a varying impact, behaviorally, on children with ASD depending on the remainder of their needs and attributes. Interestingly, we found much greater variability in the motor scores of our participants than in the adaptive behavior scores. This may be reflective of a larger variability in motor functioning across children with ASD, compared to the generally pervasive deficits in adaptive behavior, or may be an artifact of the measures used. For example, the motor coordination test was a direct assessment, however, adaptive behavior was parent-reported. We generally expect to see greater variability in direct assessments as they are dependent on the child’s performance that day, their understanding of how to perform the tasks, and their behavior. In contrast, parents are rating their child’s adaptive behavior based on multiple experiences over time, thus providing a more stable rating that is reflective of on-going challenges in the behavioral domain. Regardless, the variability in motor functioning of individuals with ASD, and how it relates to different behavioral domains, is an important area of inquiry in need of further research.

Future research should continue to disentangle the positive relationship between motor coordination and adaptive behavior found in this study, particularly given the importance of adaptive behavior for obtaining positive outcomes in children with ASD (Kanne et al., 2011). We also propose that the efficacy of motor-based interventions for improving adaptive behavior in this population be explored. Traditional behavioral and social-communicative interventions for children with ASD are often time-intensive and expensive, particularly when implemented in a community or private setting (Chasson et al., 2007). In contrast, school-based interventions may be optimal for many children with ASD as they may provide greater ecological validity; however, limited effects have been shown (Bellini et al., 2007). School-based motor skill interventions may have the potential to improve areas of functioning beyond just the motor domain. However, it is likely that the impact of motor interventions will have a varying effect across behavioral profiles of ASD. Research into the design, delivery, and outcomes of such interventions is warranted, along with an examination of the behavioral characterization of responders and non-responders to this type of intervention.

Limitations of the current study include the small sample, which limits our ability to conduct more sophisticated statistical analyses. Another limitation is that the VABS-2 is parent-reported; ideally, we would have both subjective and objective measures of the participants’ adaptive behaviors. Further, we are limited by our lack of measure of IQ, which may influence the relationship between the MABC-2 and VABS-2 found in our study. Lastly, the cross-sectional design of this study does not allow us to infer causation or disentangle the relationships between motor coordination and adaptive behavior. Future studies on this topic should employ prospective longitudinal designs and include a heterogeneous sample of participants with ASD. Strengths of the design include administration of a standardized test of motor coordination, which is also the criterion measure used to assess for DCD and other motor impairments. The multiple domains of the MABC-2 allowed us to examine interrelationships across subdomains of both motor and adaptive behaviors.

## Conclusion

The results of this study highlight the profound challenges with motor coordination and adaptive behavior experienced by school-aged children with ASD, and suggest that these challenges are interrelated. It is possible that the interrelatedness of these domains is due to the broader neurocognitive deficits present in children with ASD, however, longitudinal neuroimaging and behavioral studies are needed to disentangle these relationships.

## Ethics Statement

This study was carried out in accordance with the recommendations of Hamilton Integrated Research Ethics Board with written informed consent from all parents of participating children. All parents of participating children gave written informed consent in accordance with the Declaration of Helsinki. All child participants 8 years of age and older gave written informed assent.

## Author Contributions

EB designed the study, coordinated recruitment and data collection, carried out the data analyses, and drafted the initial manuscript. JC supervised the design and execution of all phases of the study and revised and approved the final manuscript as submitted. Both authors approved the final manuscript as submitted and agreed to be accountable for all aspects of the work.

## Conflict of Interest Statement

The authors declare that the research was conducted in the absence of any commercial or financial relationships that could be construed as a potential conflict of interest.
